# Systematic Review and Meta-analysis: The Association Between Serum Ustekinumab Trough Concentrations and Treatment Response in Inflammatory Bowel Disease

**DOI:** 10.1093/ibd/izad065

**Published:** 2023-04-18

**Authors:** Abhinav Vasudevan, Vivek Tharayil, Laura H Raffals, David H Bruining, Michelle Becker, Mohammad Hassan Murad, Edward V Loftus

**Affiliations:** Division of Gastroenterology and Hepatology, Mayo Clinic College of Medicine and Science, Rochester, MN, USA lol; Division of Gastroenterology and Hepatology, Mayo Clinic College of Medicine and Science, Rochester, MN, USA lol; Division of Gastroenterology and Hepatology, Mayo Clinic College of Medicine and Science, Rochester, MN, USA lol; Division of Gastroenterology and Hepatology, Mayo Clinic College of Medicine and Science, Rochester, MN, USA lol; Department of Pharmacy, Mayo Clinic College of Medicine and Science, Rochester, MN, USA; Robert D. and Patricia E. Kern Center for the Science of Health Care Delivery, Mayo Clinic College of Medicine and Science, Rochester, MN, USA; Division of Gastroenterology and Hepatology, Mayo Clinic College of Medicine and Science, Rochester, MN, USA lol

**Keywords:** ulcerative colitis, biologics, therapeutic drug monitoring, monoclonal antibodies, Crohn’s disease

## Abstract

**Background:**

Optimizing therapy and monitoring response are integral aspects of inflammatory bowel disease treatment. We conducted a systematic review and meta-analysis to determine whether serum ustekinumab trough concentrations during maintenance therapy were associated with ustekinumab treatment response in patients with inflammatory bowel disease.

**Methods:**

A systematic review was performed to March 21, 2022, to identify studies using MEDLINE, EMBASE, and the Cochrane library. We included studies that reported the association between serum ustekinumab trough concentrations with clinical or endoscopic remission. Outcome measures were combined across studies using the random-effects model with an odds ratio (OR) for binary outcomes of endoscopic and clinical remission.

**Results:**

We identified 14 observational studies that were included in the analysis for clinical remission (919 patients, 63% with Crohn’s disease) or endoscopic remission (290 patients, all with Crohn’s disease). Median ustekinumab trough concentrations were higher amongst individuals achieving clinical remission compared with those not achieving remission (mean difference, 1.6 ug/mL; 95% confidence interval [CI], 0.21-3.01 ug/mL). Furthermore, individuals with median serum trough concentration in the fourth quartile were significantly more likely to achieve clinical (OR, 3.61; 95% CI, 2.11-6.20) but not endoscopic remission (OR, 4.67; 95% CI, 0.86-25.19) compared with those with first quartile median trough concentrations.

**Conclusion:**

Based on the results of this meta-analysis primarily relating to patients with Crohn’s disease on maintenance ustekinumab treatment, it appears that there is an association between higher ustekinumab trough concentration and clinical outcomes. Prospective studies are required to determine whether proactive dose adjustments of ustekinumab therapy provides additional clinical benefit.

**Key Messages**

**What is already known?**

Ustekinumab is effective as induction and maintenance treatment in inflammatory bowel disease (Crohn’s disease and ulcerative colitis).

**What is new here?**

The role of therapeutic drug monitoring and ustekinumab trough concentrations to guide treatment are not established with ustekinumab maintenance therapy. This study has shown through a systematic review and meta-analysis an association between clinical remission in ulcerative colitis and Crohn’s disease with higher ustekinumab trough concentrations.

**How can this study help patient care?**

The study suggests that therapeutic drug monitoring may have a role in optimizing ustekinumab maintenance therapy in inflammatory bowel disease, and monitoring of drug levels should be considered.

## Introduction

Inflammatory bowel diseases (IBD), including Crohn’s disease (CD) and ulcerative colitis (UC), are chronic immune-mediated disorders primarily affecting the gastrointestinal tract. They commonly occur in early adulthood and require long-term medical therapy. There are now several biologic and small-molecule therapies with proven efficacy to improve long-term outcomes and demonstrate favorable safety profiles. The greater range of therapies available and the ability to achieve more stringent outcomes have resulted in a greater emphasis on proactive optimization of treatment prior to clinical deterioration. Furthermore, secondary loss of response to monoclonal antibody therapies, with the development of antidrug antibodies, can cause previously effective therapies to become ineffective due to increased drug clearance. Therapeutic drug monitoring (TDM) provides one potential mechanism to assess the need for dose escalation to improve outcomes. This approach is best established with infliximab therapy,^1,2^ but the application of TDM for other monoclonal antibodies that are not targeting tumor necrosis factor are not as established. The aim of this systematic review was to determine whether there is an association between ustekinumab trough concentrations with clinical and endoscopic outcomes in IBD. Currently, there are no systematic reviews reporting on this topic.

## Methods

### Search Strategy and Study Selection

We performed a systematic review and meta-analysis following the Preferred Reporting Items for Systematic Reviews and Meta-Analysis (PRISMA) standards,^3^ with a prespecified protocol that was registered on the PROSPERO registry (CRD42021237958). A comprehensive search of the medical literature was performed from inception up until March 21, 2022, by a medical librarian under the direction of one of the authors (A.V.) using the following resources: Ovid MEDLINEI In-Process and Other Non-Indexed Citations and Ovid MEDLI(R), Elsevier EMBASE, Cochrane Database of Systematic Reviews (CDSR), and Cochrane Central Register of Controlled Trials (CENTRAL). The bibliography of selected articles and review articles were searched to identify any further studies of relevance. Studies were identified using the following terms: *Crohn Disease*, *inflammatory bowel disease*, *colitis*, *ileitis*, *ulcerative colitis*, *regional enteritis*, *ustekinumab*, *stelara*, *CNTO1275*, and *815610-63-0*. Searches for studies were not limited by date. Only articles in English were included. The search strategy used is provided in Appendix 1.

We included randomized control trials and observational studies. Studies were included if they included adults or children with IBD who received treatment with maintenance ustekinumab and reported mean or median trough concentrations during treatment. To be included, trials had to report endoscopic or clinical remission results for patients based on the ustekinumab trough concentrations achieved. For ease of terminology, the term responder is used to describe people who achieved clinical or endoscopic remission, whereas nonresponders are individuals who do not achieve clinical or endoscopic remission, depending on the context. Where further details relating to this grouping were required, the corresponding author for the manuscript was contacted for further clarification, and the study was included if sufficient information could be obtained. Where trials reported induction and maintenance outcomes or ustekinumab concentrations, only the results on maintenance therapy were included. The primary focus of the systematic review was on maintenance therapy; therefore, studies reporting levels up to and including week 8 (induction therapy) were excluded. Given the variability in how ustekinumab trough concentrations can be reported (for example, based on quartiles, a cutoff or median concentration amongst patients in remission and not in remission), the results were combined based on the type of grouping that was performed in the study. This resulted in 3 different but related research questions that were assessed based on the reporting of drug levels: (1) remission rate in higher vs lower trough concentration (fourth vs first quartile); (2) mean (or median) trough concentrations in those achieving remission vs those not achieving remission; and (3) remission rates in high cutoff trough concentrations and lower cutoff concentrations, respectively. Where multiple analyses were performed on the same cohort in the same publication, the most comprehensive assessment was included in our analysis. The rates of antidrug antibody formation were also combined when available and reported as crude (unweighted) proportions.

### Data Extraction and Risk of Bias Appraisal

Two reviewers independently screened the titles and abstracts of identified papers based on the prespecified inclusion and exclusion criteria (A.V. and V.T.). Any discrepancies in selections were resolved by consensus amongst the investigators. Studies available only as abstracts were assessed for inclusion based on the available data provided and were included if adequate details about outcome measures and mean or median trough concentration values were available. Where multiple reports of the same patient population were published, data from the largest and most complete publication were included. The UNITI-IM trial (phase 3 ustekinumab maintenance clinical trial) had 2 large reports of outcomes,^4,5^ and the larger of these 2 reports was included.^5^ Risk of bias was assessed using the Newcastle Ottawa Scale (NOS) in which studies are judged based on 3 domains: the selection of the study groups; the comparability of the groups; and the ascertainment of the exposure and outcome of interest.^6^ We did not generate a quantitative measure for the risk of bias as recommended by recent guidance; rather, we made a global judgment based on the importance of the 3 domains to the question at hand.^7^

### Outcomes

The primary outcome of interest was the difference in serum ustekinumab trough concentrations between responders and nonresponders to therapy. We did not specify which measure of clinical remission was used nor the timing of assessment, although the use of validated disease activity index was assessed as a part of the evaluation for the risk of bias. Where multiple trough levels or outcomes were measured, the results for week 24 or closest to this were included. Additional analyses included a comparison of remission rates between the first and fourth quartile for ustekinumab levels and comparison of remission rates based on a designated cutoff value for ustekinumab levels. Planned subgroup analyses were based on disease subtype (CD vs UC) and age (adult vs pediatric) if sufficient data were available.

### Statistical Analysis and Certainty in Evidence

Binary outcomes (clinical or endoscopic remission) were analyzed using the number of events and sample size from each study group and generating an odds ratio (OR) and 95% confidence interval (CI). Odds ratio >1 implies improved response. Continuous data (trough concentration) were analyzed using the mean or median and variability measures of the trough concentration and generating a weighted mean difference and 95% CI from each study. Mean difference >0 ug/mL implies a higher value in responders. For the analysis, median and mean values were considered equivalent. The outcome measures were used across studies using the random-effects model. Between-study variance was estimated using the restricted maximum-likelihood estimator.^8^ Statistical heterogeneity was evaluated using the I^2^ statistic. Analyses were conducted using R software package (R Core Team, 2018; R: A language and environment for statistical computing. R Foundation for Statistical Computing, Vienna, Austria) as applied using *Meta Package*. The certainty in the estimates was evaluated using the GRADE approach (grading of recommendations, development, assessment, and evaluation).^9^ We evaluated publication bias by creating contour-enhanced funnel plots and conducting Egger’s regression test.

## Results

**Figure 1.**
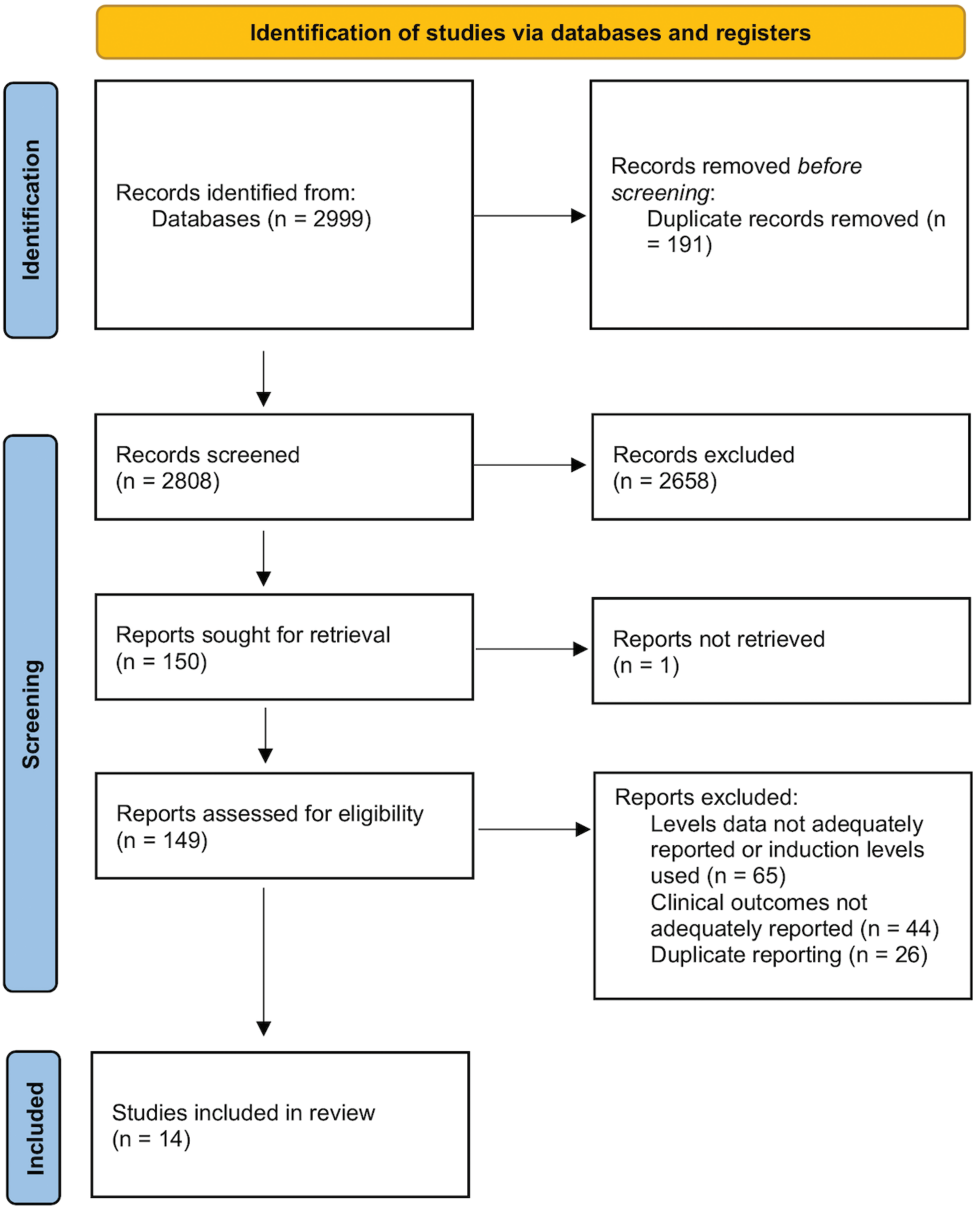
Study selection process.

The search strategy identified 2999 records of which 14 studies were included in the review for quantitative analysis. The process of study selection is depicted in Figure 1. There were 11 studies identified that were excluded, as they primarily focused on induction ustekinumab drug levels rather than maintenance therapy or the timing of drug levels was not clearly defined.^10–20^ Additionally, 4 other studies were excluded as we were not able to ascertain sufficient details of ustekinumab trough levels or the number of patients achieving remission and those not achieving remission to allow inclusion for the meta-analysis.^21–25^ The authors of 2 studies provided additional information on their study population, and this was included for calculations.^25,26^ Outcomes were reported in different forms, so these were combined based on whether studies compared the median (or mean) trough concentration (7 studies), categorized response based on trough concentrations by quartiles (2 studies) or had a prespecified cutoff trough concentration comparing responders and nonresponders (5 Studies). One included study described outcomes in UC (the UNIFI study),^27^ whereas all remaining studies reported outcomes for patients with CD. Post hoc data from the UNITI and UNIFI studies were reported as quartile data, and only 1 analysis from each trial was included in the analysis. The findings of the included studies are summarized in Table 1.

**Table 1.** Summary of included studies.

Author	Year	CD or UC	Adults or Pediatric	Number of Patients	Prior Biologic Therapies	Location and Design	UST Assay Used, Timing of Concentrations	Clinical Outcomes	Results	Comments
**Clinical Remission**										
Gomez Espin^28^	2021	CD	Adults	58	97% prior anti-TNF exposure	Retrospective, single center, Spain	Trough concentration (after week 24 of maintenance therapy), ELISA	Clinical remission (HBI <5)	Median trough concentration 2.25ug/mL in responders vs 0.65ug/mL in nonresponders	17% of patients on 4 or 6 weekly dosing
Kolar^29^	2019	CD	Adults	74	Ustekinumab median 3rd line biologic	Retrospective, single center, Czech Republic	Trough concentration (every 8 weeks), assay not stated	Clinical remission (HBI <5) at week 24	Median trough concentration 5.9ug/mL in responders vs 2.7ug/mL in nonresponders	Dosing frequency not stated
Afif^30^	2022	CD	Adults	25	90% anti-TNF exposed	Cross-sectional, multicenter (11 sites), Canada	Non-protocolized ustekinumab concentration (initiated for at least 4 weeks), ELISA	Clinical remission (HBI <5) vs mild disease (HBI 5-7) for individuals on 8 weekly ustekinumab maintenance	Median trough concentration 3.9ug/mL in responders vs 2.3ug/mL in mild disease	40% of patients were on 4 or 6 weekly dosing
Dalal^26^	2021	CD	Adults	5	100% anti-TNF exposed	Retrospective, single center, USA	Serum concentration prior to dose intensification	Corticosteroid free clinical remission (HBI <5)	Mean trough concentration 3.85ug/mL in responders vs 2.38ug/mL in nonresponders	Therapy interval was shortened following trough measurement
Verstockt^31^	2019	CD	Adults	61	95% anti-TNF exposed	Prospective, single centre, Belgium	Trough concentration (week 24), drug sensitive assay	Clinical remission (average daily stool frequency ≤2.8 and average abdominal pain score ≤1 on PRO2)	Mean trough concentration 2.27ug/mL in responders vs 2.27ug/mL in nonresponders	All received 8 weekly dosing
Adedokun^5^	2018	CD	Adults	191	54% anti-TNF exposed	Prospective, multicenter, international (27 countries)	Trough concentrations (average trough concentration through to week 44), ECLIA	Clinical remission (CDAI <150)	Response rates of 55% in quartile 1 trough concentration vs 81% in quartile 4	Included 8 and 12 weekly dosing groups
Adedokun^27^	2020	UC	Adults	318	51% prior biologic failure	Prospective, multicenter, international (24 countries)	Trough concentration (average through to week 44 of maintenance therapy)	Clinical remission (Mayo score ≤2, no subscore >1) at week 44 of maintenance therapy	Response rate 28% in quartile 1 trough concentration vs 58% in quartile 4	Included 8 and 12 weekly dosing groups
Glassner^32^	2019	CD	Not stated	46	35% dual biologic	Retrospective, single center, USA	Trough concentration, assay not stated	Clinical remission (HBI)	Using a cutoff value for ustekinumab trough concentration of 5ug/mL, response rates were 50% vs 41% in responders vs nonresponders, respectively	Dosing frequency not stated
Du^33^	2020	CD	Pediatrics	18	100% prior anti-TNF exposure	Single center, retrospective, USA	Trough concentration (maintenance)	Clinical remission (physician global assessment)	Using a cutoff value for ustekinumab trough concentration of 4.5ug/mL, response rates were 63% vs 40% in responders vs nonresponders, respectively	Standard adult dosing used
Pan^34^	2022	CD	Adults	42	50% prior anti TNF exposure	Retrospective, single center, USA	Trough concentration (maintenance therapy >12 weeks), HMSA	Clinical remission (HBI <5) in postoperative CD recurrence	Using a cutoff value for ustekinumab trough concentration of 4.5ug/mL, response rates were 33% vs 33% in responders vs nonresponders, respectively	Performed in post-operative Crohn’s. 57% received 4 weekly dosing.
Yao^35^	2021	CD	Adults	19	89% prior biologic exposure	Retrospective, single center, China	Trough concentration (week 16 or 20), ELISA	Clinical remission (CDAI < 150) at week 16/20	Using a cutoff value for ustekinumab trough concentration of 1.12ug/mL, response rates were 90% vs 78% in responder’s vs nonresponders, respectively	74% received 12 weekly dosing, 26% had 8 weekly dosing
Battat^36^	2017	CD	Adults	62	98% anti-TNF exposed	Prospective, 2 centers, Canada	Trough concentration (week 10 and week 26), HMSA	Clinical remission (HBI <5)	Using a cutoff value for ustekinumab trough concentration of 4.5ug/mL, response rates were 62% vs 71% in responder’s vs nonresponders, respectively	77% received 4 weekly dosing
**Endoscopic remission**										
Takenaka^37^	2021	CD	Adults	33	61% prior biologic exposed	Retrospective, single center, Japan	Trough concentrations (maintenance therapy)	Endoscopic remission (modified SES-CD <4)	Median trough concentration 4.2ug/mL in responders vs 1.1ug/mL in nonresponders	Dosing frequency not stated
Van den berghe^31^	2022	CD	Adults	19	79% anti-TNF exposure	Prospective, single center, Belgium	Trough concentration (week 24), ELISA	Endoscopic remission (SES-CD ≤3) at week 24	Median trough concentration 1.8ug/mL in responders vs 0.6ug/mL in nonresponders	All received 8 weekly dosing
Adedokun^5^	2018	CD	Adults	102	54% anti-TNF exposed	Prospective, multicenter, international (23 countries)	Trough concentration (average trough concentration through to week 44), ECLIA	Endoscopic remission (SES-CD ≤2)	Response rates of 8% in quartile 1 trough concentrations vs 28% in quartile 4	Included 8 and 12 weekly dosing groups
Glassner^32^	2019	CD	Not stated	19	35% dual biologic	Retrospective, single center, USA	Trough concentration, assay not stated	Endoscopic remission (scoring not mentioned)	Using a cutoff value for ustekinumab trough concentration of 5ug/mL, response rates were 14% vs 17% in responder’s vs nonresponders, respectively	Dosing frequency not stated
Pan^34^	2022	CD	Adults	42	50% prior anti TNF exposure	Retrospective, single center, USA	Trough concentration (maintenance therapy > 12 weeks), HMSA	Deep remission (endoscopic remission with an SES-CD <5 or Rutgeert’s <i2a and clinical remission)	Using a cutoff value for ustekinumab trough concentration of 4.5ug/mL, response rates were 28% vs 17% in responder’s vs nonresponders, respectively	Performed in post-operative CD. 57% received 4 weekly dosing
Yao^35^	2021	CD	Adults	19	89% prior biologic exposure	Retrospective, single center, China	Trough concentration (week 16 or 20), ELISA	Endoscopic remission (SES-CD ≤2)	Using a cutoff value for ustekinumab trough concentration of 1.12ug/mL, response rates were 70% vs 11% in responder’s vs nonresponders, respectively	74% received 12 weekly dosing, 26% had 8 weekly dosing
Battat^36^	2017	CD	Adults	56	98% anti-TNF exposed	Prospective, 2 centers, Canada	Trough concentration (week 10 and week 26), HMSA	Endoscopic remission (SES-CD ≤2)	Using a cutoff value for ustekinumab trough concentration of 4.5ug/mL, response rates were 28% vs 11% in responder’s vs nonresponders, respectively	77% received 4 weekly dosing

Abbreviations: UST, ustekinumab; UC, ulcerative colitis; CD, Crohn’s disease; ELISA, enzyme-linked immunosorbent assay; HBI, Harvey-Bradshaw Index; Anti-TNF, tumor necrosis factor alpha inhibitor; HMSA, homogenous mobility shift assay; SES-CD, Simple Endoscopic Score for Crohn’s disease; ECLIA, electrochemiluminescence immunoassay; PRO2, weighted patient reported outcome.

### Comparison of Trough Concentrations Between Remission and Active Disease

**Figure 2.**
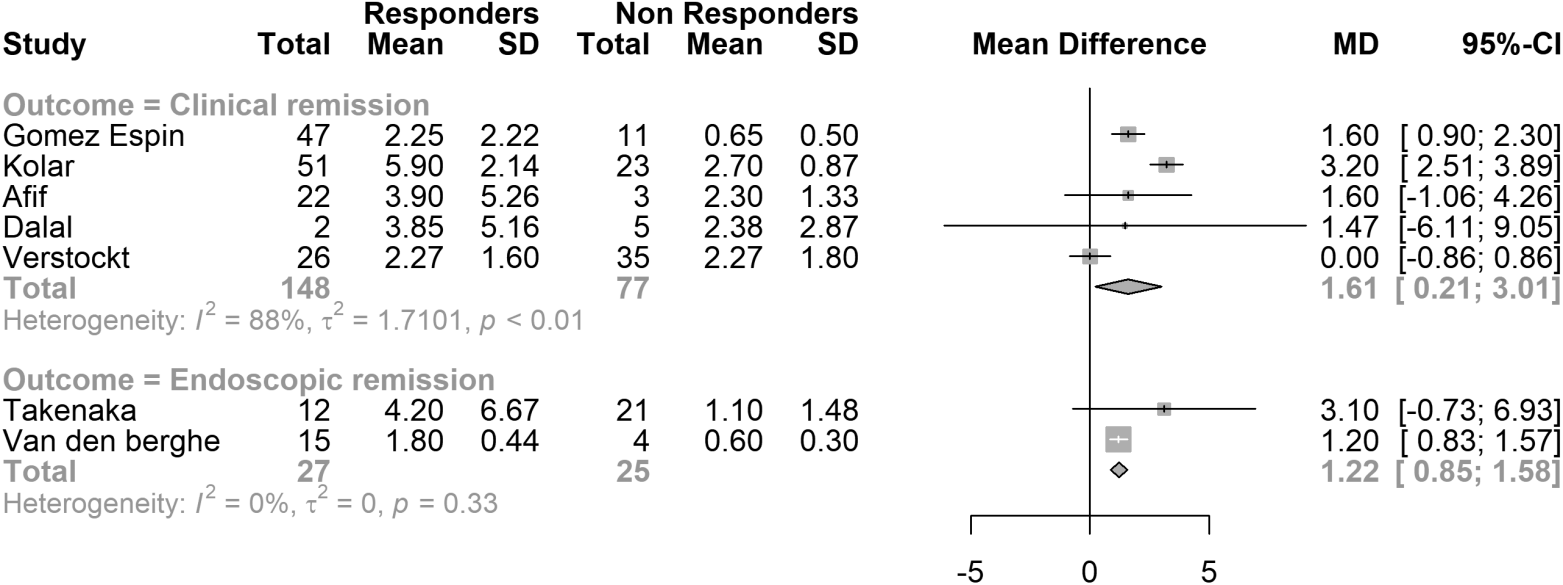
Meta-analysis of mean ustekinumab trough concentration for remission and nonremission.

There were 5 studies in adults with CD that reported mean or median ustekinumab trough concentrations for patients in clinical remission and compared those concentrations to those not achieving remission. The mean trough concentration of ustekinumab was higher in patients in clinical remission compared with nonremitters, with a mean difference of 1.61 ug/mL (95% CI, 0.21-3.01 ug/mL). There were 2 studies in adults with CD that reported mean or median ustekinumab trough concentrations for patients in endoscopic remission. The mean trough concentration of ustekinumab was higher in endoscopic remission compared with those not in remission, with a mean difference of 1.22 ug/mL (95% CI, 0.85-1.58 ug/mL). There was considerable statistical heterogeneity in both analyses. The results are depicted in Figure 2.

### Remission Based on Ustekinumab Trough Concentration Quartiles

**Figure 3.**
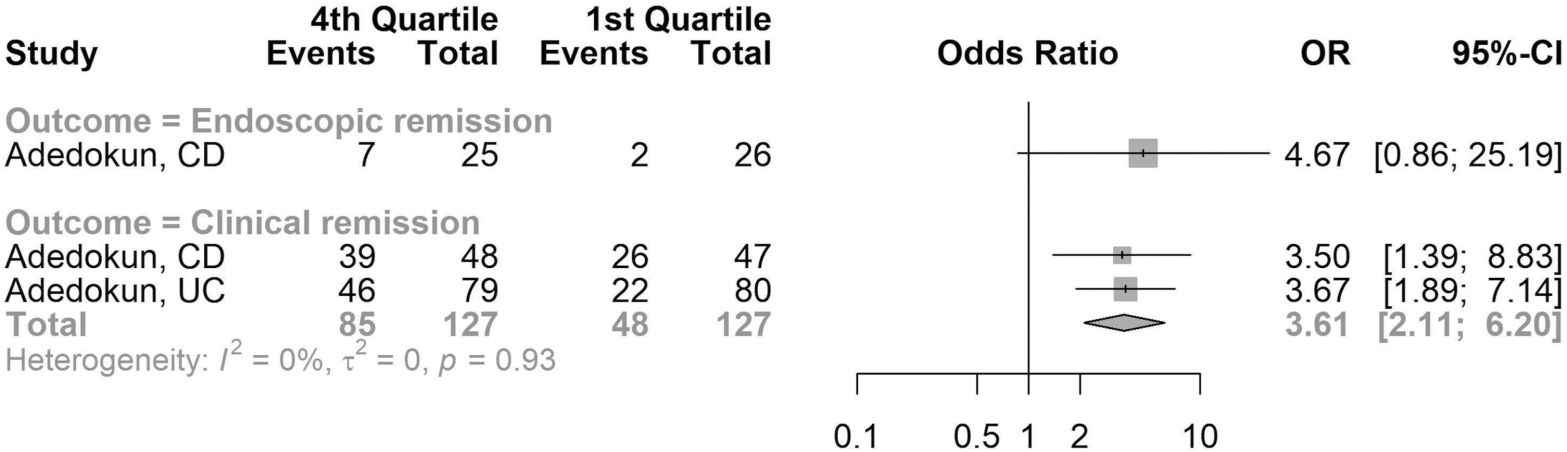
Meta-analysis of clinical and endoscopic remission based on ustekinumab trough concentration quartiles in Crohn’s disease (CD) and ulcerative colitis (UC).

Two studies reported rates of clinical remission rates based on quartiles of ustekinumab trough concentration in adults (1 study in CD, 1 study in UC).^5,27^ Trough concentrations in the fourth quartile were associated with higher clinical remission rates than concentrations in the first quartile (OR, 3.61; 95% CI, 2.11-6.20). The analysis did not demonstrate important statistical heterogeneity. The results were consistent between CD and UC. One study reported rates of endoscopic remission rates at week 24 based on quartiles of ustekinumab trough concentration in adults with CD.^5^ There was a numerically higher endoscopic remission rate amongst individuals with the highest quartile levels compared with the lowest quartile levels, but this did not reach statistical significance (OR, 4.67; 95% CI, 0.86-25.19). The results are depicted in Figure 3.

### Comparison of Remission Rate Based on Cutoff Value for Ustekinumab Concentrations

**Figure 4.**
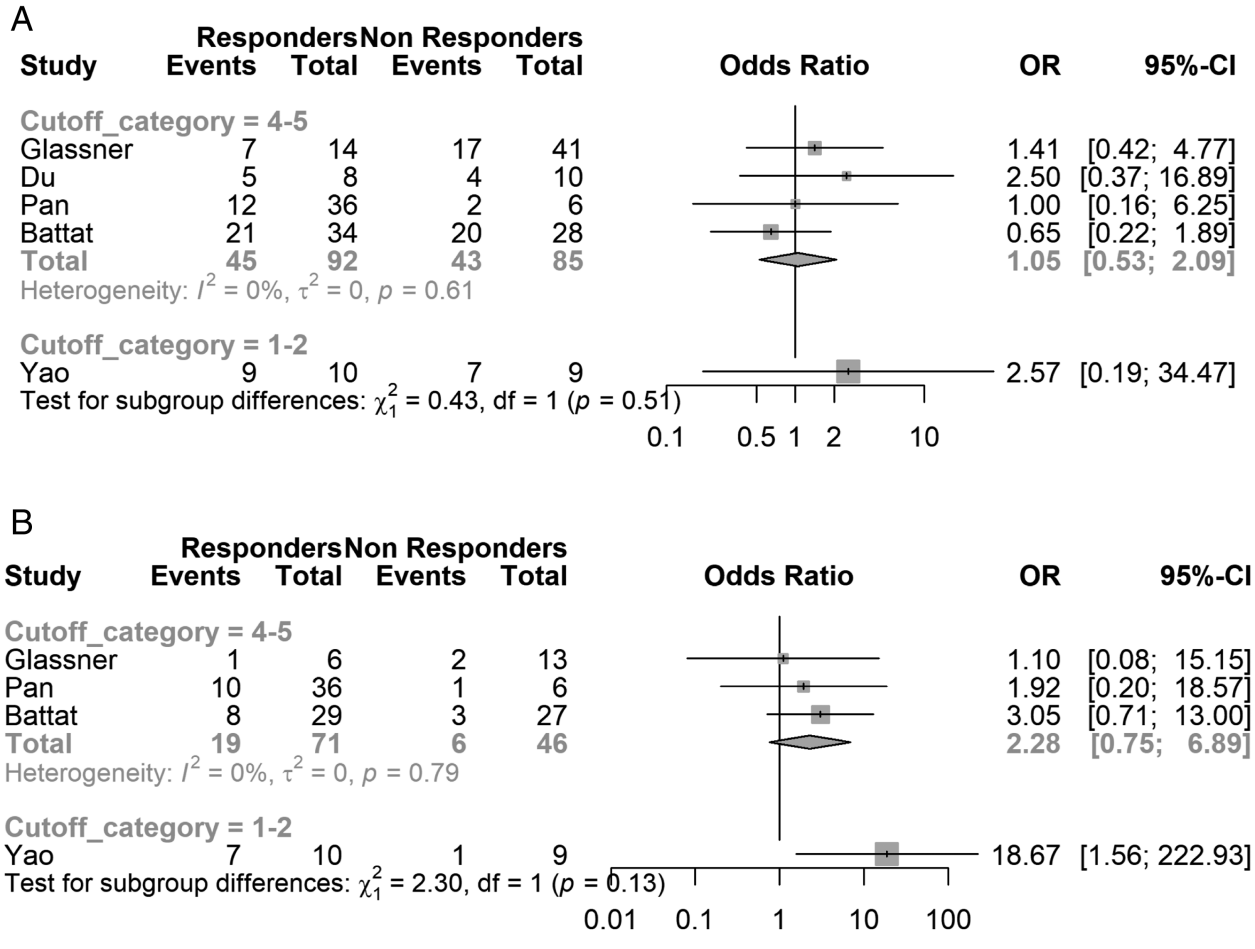
Meta-analysis of clinical remission rates based on ustekinumab trough concentration cutoff value in Crohn’s disease (CD) for (A) clinical remission and (B) endoscopic remission.

Five studies reported rates of clinical remission in CD based on a designated cutoff value for ustekinumab trough concentrations, with 1 study being in pediatric population. Four studies reported rates of endoscopic remission in CD based on a designated cutoff value for ustekinumab trough concentrations, with 3 studies being in adult patients and 1 study not specifying the age group studied. Studies were grouped based on whether the defined cutoff trough concentration was between 1 and 2 ug/mL or between 4 and 5 ug/mL to allow combining of results. Comparison of cutoffs that ranged 1 to 2 ug/mL with those that ranged 4 to 5 ug/mL did not demonstrate a significant difference in clinical remission rate or endoscopic remission rate (Figures 4A and 4B). Additionally, within each cutoff category, no significant difference is noted between responders and nonresponders. These comparisons were underpowered due to the small number of studies included in each cutoff category.

### Immunogenicity of Ustekinumab

There were 7 reports of antidrug antibodies from post hoc analyses of randomized controlled trials including the UNITI and UNIFI studies and their long-term extensions, and the prevalence of patients positive for antidrug antibodies ranged between 4.2% and 5.6%.^38–44^ Combined data from 13 observational studies reported antidrug antibodies in 9 of 751 (1.2%) patients.^14,28,35–37,45–53^

### Risk of Bias and Certainty of Evidence

Risk of bias indicators using the NOS instrument are summarized in Table 2. Overall, the risk of bias was moderate, with most studies having adequate study selection approach and ascertainment of exposure and outcomes. However, the comparability domain of NOS was not satisfied for most studies since there was no adequate adjustment for confounders. The GRADE certainty in the estimates was very low due to the nonrandomized nature of the analysis and the imprecision caused by having a small number of patients and small number of studies. Data were insufficient to conduct meaningful subgroup analyses. Inspection of funnel plot and conducting Egger’s regression test for clinical and endoscopic remission analysis (7 studies included) did not suggest the presence of publication bias (Appendix 2).

**Table 2.** Risk of bias.

Gomez Espin	Kolar	Afif	Dalal	Takenaka	Van den berghe	Adedokun^a^	Adedokun ^b^	Glassner	Du	Pan	Yao	Battat	Verstockt	
+	-	+	+	+	-	+	+	-	+	-	+	+	+	Selection case definition
+	-	+	+	+	-	+	+	-	+	-	+	+	+	Representativeness of cases
+	-	+	+	+	-	+	+	+	+	-	+	+	+	Selection of controls/ascertainment of exposure
-	-	+	-	+	+	+	+	-	-	+	+	+	+	Definition of controls/Demonstration that active disease was present at baseline
+	+	+	+	+	+	+	+	+	+	+	+	+	+	Comparability for remission
+	-	-	+	+	+	+	+	+	+	+	+	+	+	Assessment of exposure/outcome
-	+	-	+	-	+	+	+	+	+	+	+	+	+	Long enough follow up or same method of ascertainment for cases/controls
-	-	-	+	-	+	+	+	-	+	-	-	+	+	Adequacy of follow up or non response rate

- Indicates high risk of bias.

+ Indicates low risk of bias.

^a^UNITI-IM study.^5^

^b^UNIFI study.^27^

## Discussion

Monoclonal antibody therapies inhibiting interleukin-23 and interleukin-12, including ustekinumab, are becoming an increasingly important class of therapies in treating IBD, so understanding mechanisms to optimize this class of treatment are needed. This systematic review and meta-analysis attempted to determine whether currently available evidence supported a relationship between ustekinumab trough concentration and clinical or endoscopic outcomes in IBD. The cross-sectional data available suggest a significant association between higher ustekinumab trough concentrations and a higher rate of clinical remission in CD and UC. Ustekinumab trough levels were numerically higher among those in endoscopic remission, but this difference did not reach statistical significance. The included studies evaluating endoscopic remission were only in patients with CD, and the lower number of endoscopic assessments may make this analysis underpowered. Similarly, the relative paucity of data did not permit meaningful additional subgroup analyses to determine differences based on age groups (pediatric or adult) or disease subtype (CD or UC).

The prevalence of antidrug antibodies on maintenance therapy with ustekinumab appears to be lower than that reported with infliximab. Our analysis found reports of 4.2% to 5.6% based on the large clinical trials, whereas reports of infliximab antidrug antibodies have been reported in a previous meta-analysis to be as high as 12.4% on maintenance therapy and up to 45.6% with episodic dosing.^54^ The presence of antidrug antibodies against ustekinumab in observational studies was lower than that in the clinical trials. Thus, the clinical importance of immunogenicity with ustekinumab therapy may be lower than with anti-TNF therapy, particularly infliximab. We did not distinguish between individuals on combination immunomodulators and those on ustekinumab monotherapy, so the need for concomitant therapy could not be established from our study. Ustekinumab antibody detection was generally higher in randomized control trials compared with real-world studies. This may relate to differences in the assay used to detect antibodies, including drug-sensitive, drug-tolerant, and drug-specific assays rather than actual differences in antidrug antibody levels, and this should be considered when interpreting the findings.

There is not a universally accepted method for reporting trough concentration and clinical outcomes. Studies evaluating the role of ustekinumab serum trough concentrations have used a variety of reporting methods, such as quartiles, a prespecified cutoff or mean or median values in responders and nonresponders. This likely reflects the uncertainty surrounding interpreting serum trough concentrations, particularly in the absence of an established therapeutic range. Similar issues were noted when initial recommendations surrounding infliximab trough concentrations were made. As time has progressed, further studies have noted the need for higher trough concentrations to achieve more rigorous therapeutic end points such as histological remission when using infliximab.^55^ It is likely that further refining of the target ustekinumab trough concentration will occur as more data become available. Given the differences in reporting levels and the lack of apparent difference between a higher and lower cutoff values and clinical outcomes, we were not able to define a set cutoff value that was associated with better outcomes, and it may also suggest that a change in ustekinumab trough concentrations may be important in improving outcomes; hence, a trial of higher doses of therapy could be considered in patients who have not responded to standard dosing.

Although our analysis noted a correlation between higher serum ustekinumab trough concentrations and improved clinical outcomes, there is limited evidence to support proactive optimization of therapy to achieve a higher serum concentration resulting in improved clinical outcomes. Prior retrospective studies have shown an improvement in clinical outcomes following dose interval shortening of ustekinumab therapy,^56–58^ although these studies did not evaluate the role of therapeutic drug monitoring in guiding this decision. A small prospective study evaluating ustekinumab trough concentrations both before and after either dose interval shortening or re-induction with ustekinumab found that individuals in complete remission (clinical and biochemical remission) following treatment escalation had higher mean post-treatment ustekinumab trough concentration compared with those who did not achieve complete remission (13.04 ug/mL vs 8.57 ug/mL; *P* = .03).^59^ Similarly, a retrospective study of 44 patients with active Crohn’s disease on maintenance ustekinumab therapy noted that individuals achieving endoscopic remission following dose escalation to 4 therapies per week had higher concentrations than those who did not achieve endoscopic remission (6.90 vs 4.29 mg/L; *P* = .025).^60^ Further studies are needed specifically evaluating the role of therapeutic drug monitoring and proactive dose escalation of ustekinumab therapy to achieve clinical outcomes, and these are anticipated to be available in the future with ongoing trials being performed to address this issue (NCT04245215).

The strength of this study is the ability to provide a pooled analysis from both observational and post hoc analyses regarding the association between ustekinumab concentrations and clinical and endoscopic remission. The main limitation in the study was lack of randomized trials available to specifically address this clinical question, so there is the risk of bias, in addition to the relatively small number of published studies. Additionally, there were a number of different assays used to assess drug levels, and it has previously been suggested that the agreement between different assay types is poor.^61^ There was not sufficient data available in this review to make an assessment regarding differences between the different types of assays used, and a noted limitation in this analysis is pooling such heterogenous assays, which may limit clear conclusions particularly relating to specific target cutoff values. We only assessed serum levels and did not evaluate tissue drug levels, and reports evaluating this have suggested that serum levels rather than tissue levels correlate with biochemical response.^62^ Additionally, although our analysis did not suggest the presence of publication bias, the number of studies included in the analysis was quite small, and hence, publication bias remains possible.

## Conclusion

For patients being treated with ustekinumab therapy for IBD, it appears that there is an association between higher ustekinumab trough concentrations and improved outcomes, with stronger evidence to support better clinical outcomes than endoscopic outcomes. Further trials are needed to clarify the role of proactive therapeutic drug monitoring and dose adjustment of ustekinumab therapy to achieve a target trough concentration.

## Supplementary Material

izad065_suppl_Supplementary_Appendix

## Data Availability

Data, analytic methods, and study materials will be made available to other researchers upon written request to the corresponding author.
