# Supplementary material for: Systematic Review and Meta-analysis: The Association Between Serum Ustekinumab Trough Concentrations and Treatment Response in Inflammatory Bowel Disease
Source: Inflamm Bowel Dis. 2023 Apr 18;30(4):660–70. doi: 10.1093/ibd/izad065 (PMC10988107; doi:10.1093/ibd/izad065)
Supplement: izad065_suppl_Supplementary_Appendix [file izad065_suppl_supplementary_appendix.docx]

**Appendix 1. Actual Search Strategies**

**OVID**

Database(s): **Ovid MEDLINE(R) 1946 to Present and Epub Ahead of Print, In-Process & Other Non-Indexed Citations and Ovid MEDLINE(R) Daily, EBM Reviews - Cochrane Central Register of Controlled Trials**March 2022**, EBM Reviews - Cochrane Database of Systematic Reviews**2005 to March 22, 2022**, Embase**1974 to 2021 March 22 2022
Search Strategy:

| **#** | **Searches** |
| --- | --- |
| 1 | exp Inflammatory Bowel Diseases/ |
| 2 | exp Crohn disease/ |
| 3 | exp ulcerative colitis/ |
| 4 | enteritis/ |
| 5 | ("cleron disease" or "colitis ulcerativa" or "colitis ulcerosa" or crohn* or enteritis or ileocolitis or ileitis or "inflammatory bowel disease*" or "inflammatory enteropath*" or "mucosal colitis" or "regional enterocolitis" or "regional enteritis" or "regional ileitis" or "regional ileitides" or "ulcerative colitis" or "ulcerative colorectitis" or "ulcerative procto colitis" or "ulcerative proctocolitis" or "ulcerous colitis").ti,ab,hw,kw. |
| 6 | or/1-5 |
| 7 | Ustekinumab/ |
| 8 | (ustekinumab or stelara or CNTO1275 or CNTO-1275 or "CNTO 1275" or "815610-63-0").ti,ab,hw,kw. |
| 9 | 7 or 8 |
| 10 | 6 and 9 |
| 11 | 10 not ((exp animals/ or exp nonhuman/) not exp humans/) |
| 12 | limit 11 to english language [Limit not valid in CDSR; records were retained] |
| 13 | remove duplicates from 12 |

**SCOPUS**

| 1 | TITLE-ABS-KEY ("cleron disease" or "colitis ulcerativa" or "colitis ulcerosa" or crohn* or enteritis or ileocolitis or ileitis or "inflammatory bowel disease*" or "inflammatory enteropath*" or "mucosal colitis" or "regional enterocolitis" or "regional enteritis" or "regional ileitis" or "regional ileitides" or "ulcerative colitis" or "ulcerative colorectitis" or "ulcerative procto colitis" or "ulcerative proctocolitis" or "ulcerous colitis") |
| --- | --- |
| 2 | TITLE-ABS-KEY (ustekinumab or stelara or CNTO1275 or CNTO-1275 or "CNTO 1275" or "815610-63-0") |
| 3 | 1 and 2 |
| 4 | INDEX(embase) OR INDEX(medline) OR PMID(0* OR 1* OR 2* OR 3* OR 4* OR 5* OR 6* OR 7* OR 8* OR 9*) |
| 5 | 3 not 4 |
| 6 | DOCTYPE(ed) OR DOCTYPE(bk) OR DOCTYPE(er) OR DOCTYPE(no) OR DOCTYPE(sh) OR DOCTYPE(ch) |
| 7 | 5 not 6 |
| 8 | LANGUAGE(english) |
| 9 | 7 and 8 |

**Web of Science**

| 1 | TS=("cleron disease" or "colitis ulcerativa" or "colitis ulcerosa" or crohn* or enteritis or ileocolitis or ileitis or "inflammatory bowel disease*" or "inflammatory enteropath*" or "mucosal colitis" or "regional enterocolitis" or "regional enteritis" or "regional ileitis" or "regional ileitides" or "ulcerative colitis" or "ulcerative colorectitis" or "ulcerative procto colitis" or "ulcerative proctocolitis" or "ulcerous colitis") |
| --- | --- |
| 2 | TS=(ustekinumab or stelara or CNTO1275 or CNTO-1275 or "CNTO 1275" or "815610-63-0") |
| 3 | 1 and 2 |
| 4 | PMID=(0* or 1* or 2* or 3* or 4* or 5* or 6* or 7* or 8* or 9*) |
| 5 | 3 not 4 |
| 6 | LANGUAGE: (English) |
| 7 | 5 and 6 |

**Appendix 2**

**Funnel plot and Egger regression test evaluating for publication bias for clinical remission and endoscopic remission studies**
